# A 57-Year-Old With a History of Pulmonary TB Presents With Chronic Hemoptysis and Cavitary Lung Lesion

**DOI:** 10.1016/j.chpulm.2026.100283

**Published:** 2026-07-14

**Authors:** Jack Tomen, Subramanya Shyam Ganti, Natalie Aulicino, Michael Aulicino, Frank Baciewicz, Ayman O. Soubani, Amina Pervaiz

**Affiliations:** a Department of Internal Medicine, Division of Pulmonary, Critical Care and Sleep Medicine, Wayne State University School of Medicine, Detroit, MI; b Department of Internal Medicine, Division of Pulmonary Critical Care Appalachian Regional Health Care Hospital, Harlan, KY; c Department of Pathology, Detroit Medical Center, Wayne State University School of Medicine, Detroit, MI

## Abstract

A 57-year-old man with a history significant for *Mycobacterium tuberculosis* infection presented to the emergency department for a 5-month history of low-volume hemoptysis, fatigue, and unintentional weight loss of 12 lb. He was treated twice in the past for pan-sensitive TB with a standard regimen of isoniazid, rifampin, pyrazinamide, and ethambutol for 12 months.

Over the past 5 months, his hemoptysis had progressively worsened, reaching approximately one-quarter of a cup per day. However, on the day before presentation, he experienced an episode of massive hemoptysis amounting to one-half of a cup of bright red blood, prompting him to present to the emergency department.

His history included post-TB bronchiectasis diagnosed after the completion of his most recent TB treatment. He had worked as a seasonal landscaper for nearly 30 years with frequent soil exposure and reported daily marijuana smoking for approximately 20 years. He denied prior incarceration or travel to TB-endemic regions and had lived in a metropolitan area throughout his life.

## Physical Examination Findings

On presentation, his vital signs were notable for temperature of 37.4 °C, heart rate of 105 beats/min, BP of 118/64 mm Hg, respiratory rate of 16 breaths/min, and oxygen saturation of 99% on room air. He appeared cachectic with significant temporal muscle wasting. He was in no acute distress, and lung auscultation revealed decreased breath sounds in the left lung field. The remaining physical examination was unremarkable.

## Diagnostic Studies

Initial laboratory analysis showed microcytic anemia with a hemoglobin level of 9.5 g/dL., MCV level of 80.2 fL, and normal leukocyte count with a normal differential; HIV testing was negative. Additional tests did not reveal evidence of other autoimmune diseases or immunosuppressive medication use. Chest radiograph demonstrated chronic fibrocystic changes in the upper zone of the left hemithorax with volume loss and ipsilateral shift of the heart and trachea (Fig 1). CT scan of the chest showed severe bronchiectasis with interstitial airspace opacities in the left upper and lower lobes with a cavitary lesion in the left upper lobe containing a soft tissue density (Fig 2). The right lung showed tree-in-bud opacities in the right upper lobe (Fig 2). These findings are suspicious for chronic cavitary pulmonary aspergillosis (CCPA) secondary to the patient’s history of cavitary pulmonary TB with resultant fungal colonization. The progression to massive hemoptysis and new right-sided nodular opacities on CT imaging are concerning for progression to invasive disease.

**Figure 1:**
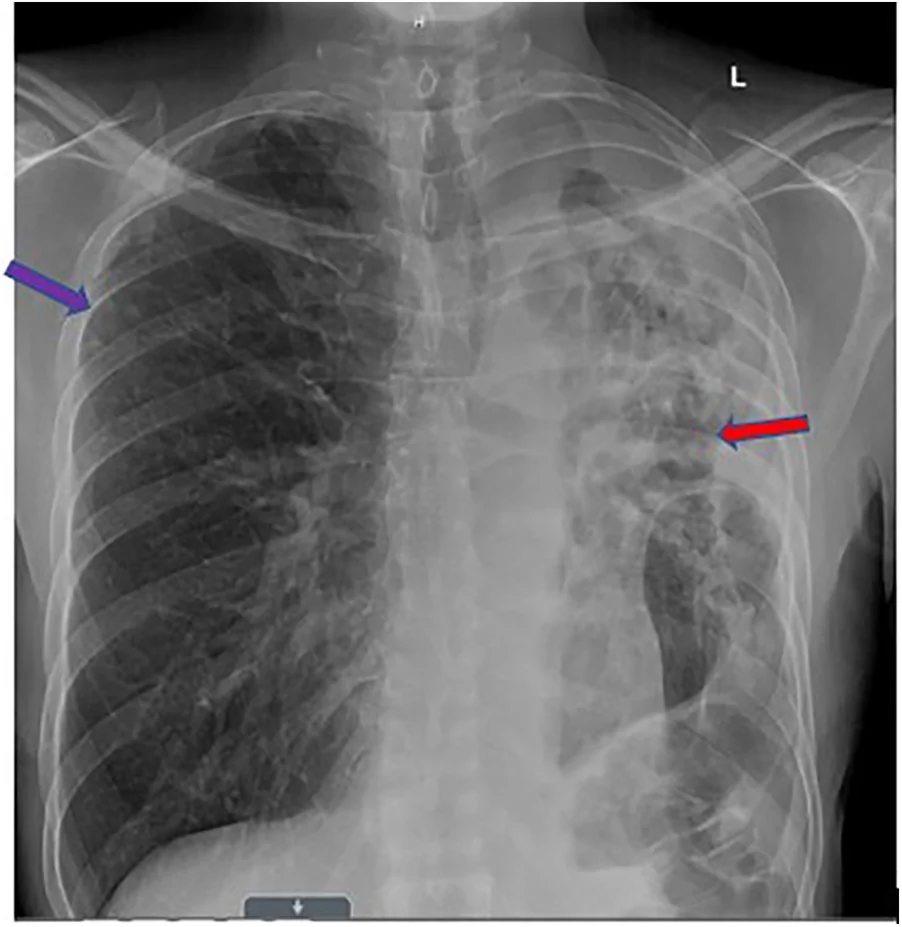
Chest radiograph, posterior-anterior projection, showing left-sided bronchiectasis changes and volume loss (red arrow) and tree-in-bud nodularity in the right upper lung field (purple arrow).

**Figure 2:**
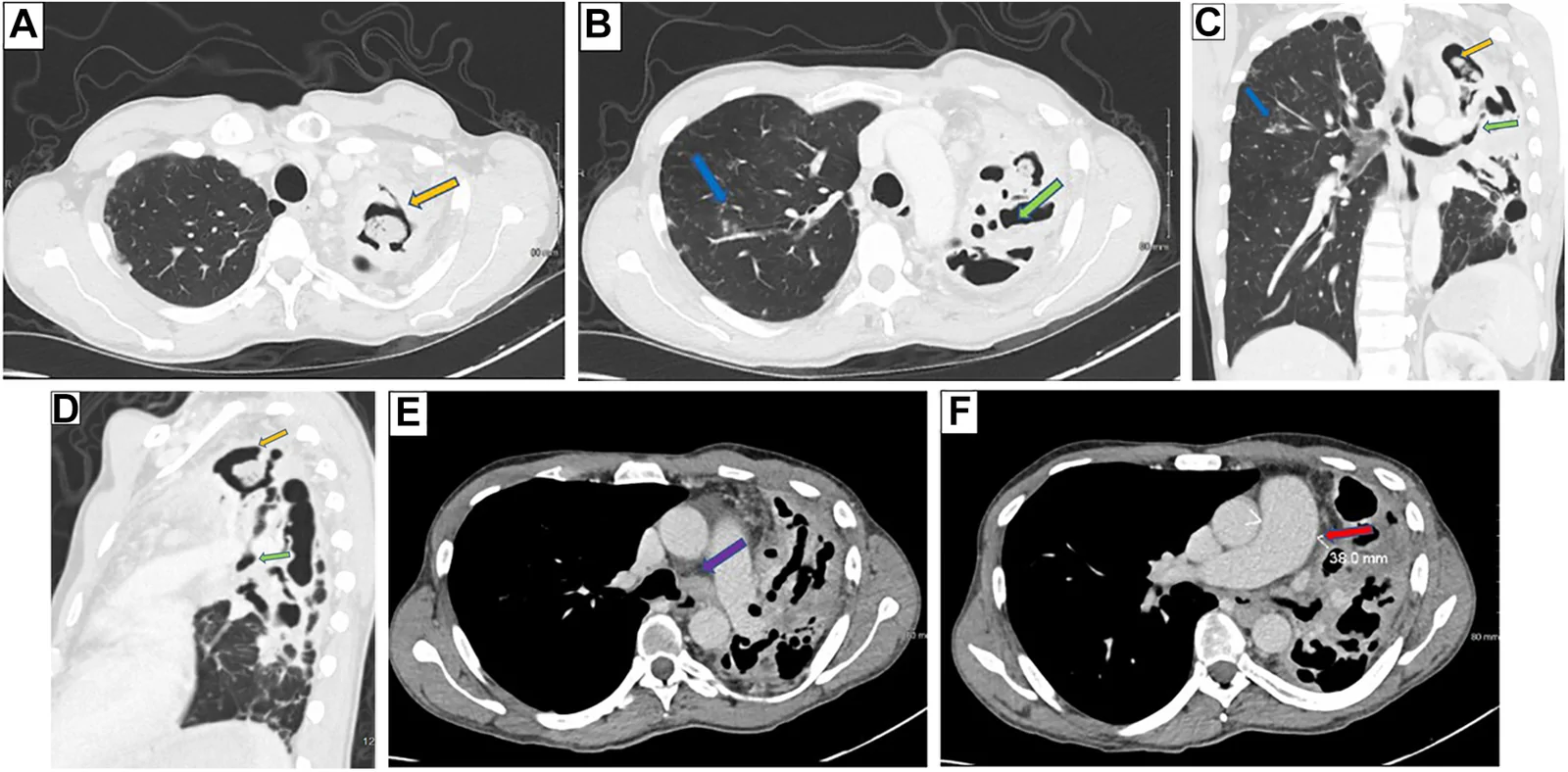
A-D, CT scan of the chest, lung window, axial, coronal, and sagittal views showing the aspergilloma (yellow arrow), tree-in-bud nodularity in the right upper lobe (blue arrow), and significant bronchiectatic changes in the left upper lobe with volume loss (green arrow, prominent on the coronal view). E, F, Mediastinal window showing enlarged lymph nodes (purple arrow) and marked dilation of the pulmonary trunk (red arrow).

Respiratory cultures were negative; therefore, bronchoscopy was performed for diagnostic evaluation of the cavitary and nodular lesions to exclude malignancy, nontuberculous mycobacterial (NTM) infection, and fungal disease, and to obtain microbiological confirmation. The patient underwent bronchoscopy with bronchoalveolar lavage (BAL) from the anterior segment of the right upper lobe (RB3). BAL testing was positive for *Aspergillus* antigen, whereas bacterial, acid-fast bacilli, and fungal cultures were finalized as negative. The patient’s symptoms of progressive hemoptysis and weight loss, worsening destructive cavitary changes on imaging, positive *Aspergillus* serology (galactomannan and elevated serum IgG), exclusion of TB and NTM on BAL and surgical cultures, and histopathologic evidence of *Aspergillus* within and adjacent to lung tissue helped in establishing the diagnosis.

*What is the diagnosis?*

*Diagnosis:* Hemoptysis because of CCPA

## Discussion

*Aspergillus* species are responsible for a spectrum of pulmonary infections. The most common form of aspergillus disease is an aspergilloma, in which a fungus ball forms in a preexisting pulmonary cavity, such as those formed by a previous TB infection. In most cases, patients are asymptomatic but may present with hemoptysis. This is often mild, but in rare cases may be life-threatening or require intervention. The etiology of hemoptysis is caused by erosion of blood vessels within the cavity wall.

In contrast to a simple aspergilloma, CCPA is a progressive and more destructive entity characterized by systemic and respiratory symptoms and the presence of ≥ 1 pulmonary cavities. Unlike the typically stable course of an aspergilloma, CCPA shows radiologic progression, including new or enlarging cavities, increasing pericavitary inflammation, or fibrosis over time.

Diagnosis of CCPA relies on clinical evaluation, laboratory tests, and imaging studies. Sputum cultures are frequently negative; only 19% of sputum samples showed fungal elements on gram stain, whereas 76% were culture positive. BAL samples tested for galactomannan have a sensitivity of 85.7% and a specificity of 76.3% and are superior to sputum and fungal cultures in identifying fungus. MTB and NTM are important conditions to exclude in patients with an aspergilloma because they may have similar presentations or can coexist.

This makes lavage samples for galactomannan an important tool in the diagnosis of pulmonary aspergillosis because imaging alone is not sufficient given the broad infectious differential diagnosis related to chronic pulmonary aspergillosis.

Another important test is serum *Aspergillus* IgG, which demonstrates a sensitivity of 80% to 92% and a specificity of 69% to 73%. In cases of chronic cavitary aspergillosis, serum *Aspergillus*-specific IgG was positive in 96.2% of patients. Although not diagnostic of an active infection, its role is in providing evidence of aspergillus exposure, which supports the final diagnosis.

Treatment of CCPA is often multimodal, with medical/antifungal therapy and surgical intervention depending on the degree of hemoptysis. Triazole antifungal therapy is recommended in patients with pulmonary symptoms of cough, hemoptysis, or weight loss. Of the patients treated with itraconazole, 67% experienced radiographic or symptomatic improvement, compared with 35.7% in the placebo group. The Infectious Diseases Society of America recommends treatment with oral antifungal therapy for a minimum of 6 months.

Other interventions for treating recurrent or ongoing hemoptysis are tranexamic acid, bronchial artery embolization (BAE), and surgical resection. Embolization of the bronchial artery supplying the cavity may be considered for life-threatening hemoptysis as a bridge to definitive surgical therapy. Recurrence of hemoptysis is common with intraarterial embolization and should not be considered definitive therapy, with 15% of patients having recurrence within 1 month after embolization, increasing to around 30% over the long term. Most recurrences occur within the first 9 months.

A lobectomy is the most common procedure; however, other surgical options may be considered based on the extent of the disease and the patient’s clinical status. Surgical intervention carries a risk of prolonged air leaks and fungal empyema but results in cessation of hemoptysis for 83% of patients. Culture data obtained intraoperatively can be used to help establish a definitive diagnosis and treatment plan. Postoperatively, a 3-month course of antifungal therapy is recommended, followed by repeat imaging to assess residual disease. Surgical intervention often leads to the resolution of hemoptysis in patients with CCPA, with one study reporting that 83% of patients experienced cessation of hemoptysis after surgery.

## Clinical Course

The patient was not a candidate for BAE because of the presence of extensive lung disease and the absence of a clear bleeding source. Because of severe left lung destruction, a simple resection of the aspergilloma was not feasible; therefore, the patient underwent an emergent left total pneumonectomy. Pathology of the resected left lung revealed acute and chronic granulomatous inflammation with *Aspergillus* in the left lung cavity without evidence of invasive aspergillosis, consistent with CCPA (Fig 3). He completed 6 months of antifungal therapy, initially with voriconazole and then with isavuconazonium.

**Figure 3:**
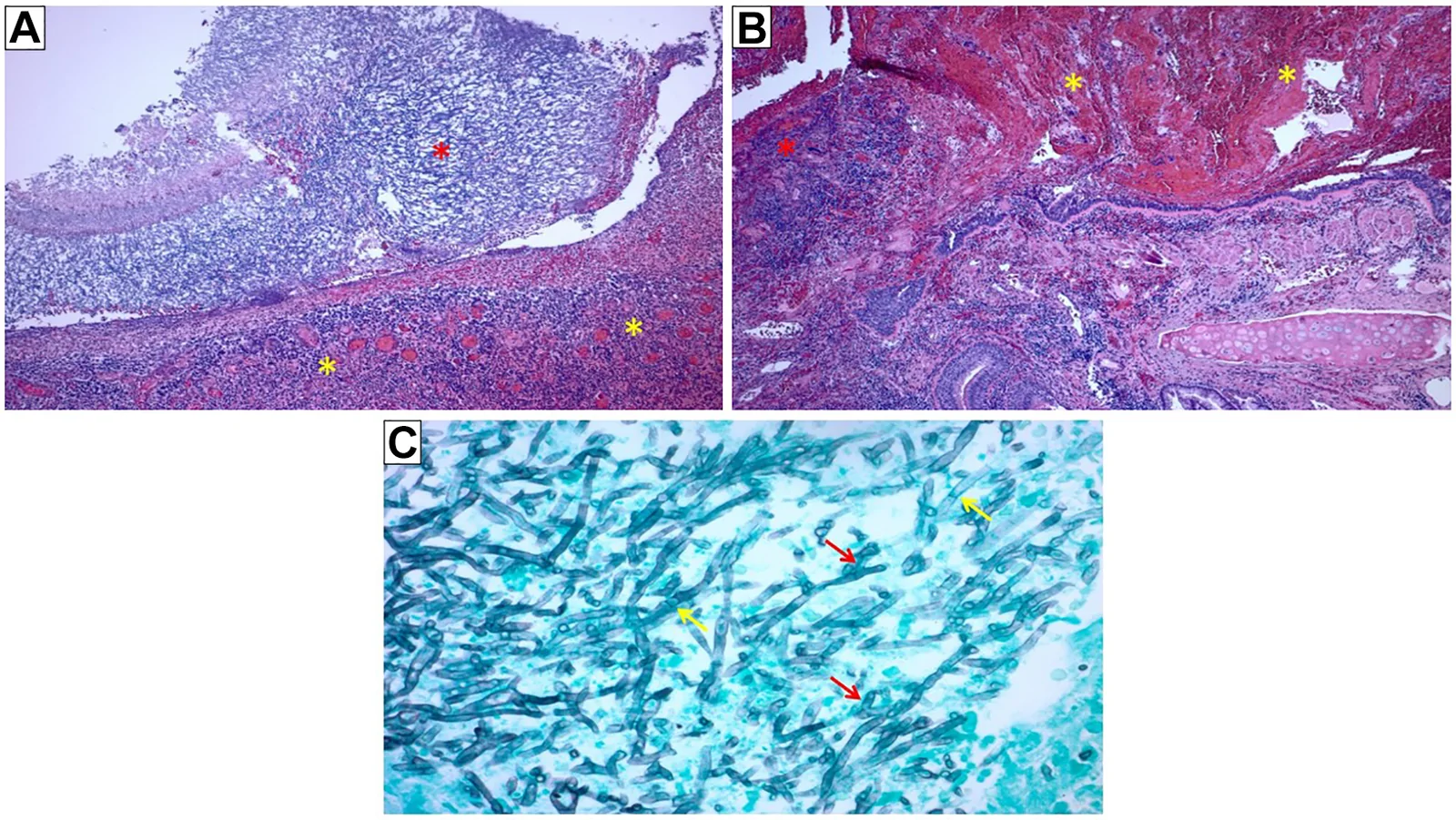
A-C, A cavitary space is present in the upper portion of the figure containing fungal elements admixed with inflammatory debris (red asterisk). The cavity wall and surrounding lung demonstrate marked chronic inflammation, fibrosis, and remodeled lung (yellow asterisks) (hematoxylin and eosin stain, ×10 magnification). The cavity wall is seen in the left portion of the image (red asterisk) and is noted to be eroding into a bronchial lumen with attached organizing blood clot (yellow asterisks) (hematoxylin and eosin stain, ×10 magnification). Fungal organisms are present within the cavity without evidence of angioinvasion; fungal hyphae demonstrate acute-angle, dichotomous branching (red arrows) and occasional septation (yellow arrows) consistent with Aspergillus species (Grocott’s methenamine silver stain, ×20 magnification).

At follow-up visits, the patient reported no limitations in daily activities. He had no additional episodes of hemoptysis. CT chest scan after pneumonectomy revealed expected postoperative changes, and the resolution of tree-in-bud nodularity in the right upper lobe (Fig 4).

**Figure 4:**
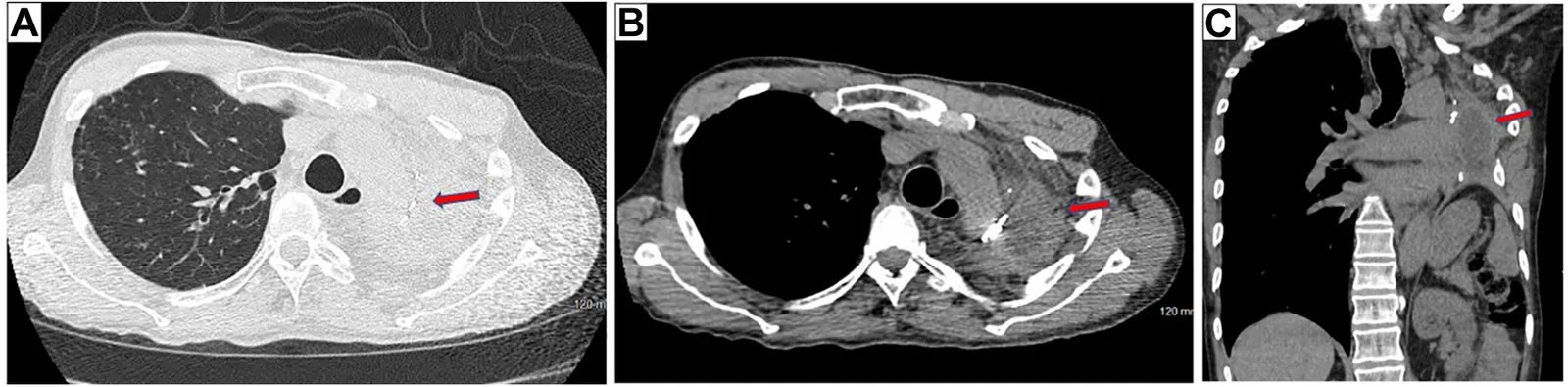
A-C, CT scan of the chest, lung window, axial and coronal views, showing the left pneumonectomy and left pleural cavity filled with fluid that is circumferentially encased by diffusely thickened pleura (red arrow).

## Clinical Pearls

1. *BAL galactomannan testing is a valuable diagnostic tool for identifying chronic pulmonary aspergillosis.*
2. *BAE should be viewed as a temporary and stabilizing intervention for treatment of hemoptysis secondary to an aspergilloma. It frequently recurs after the procedure.*
3. *Surgical resection is the definitive treatment for patients with recurrent hemoptysis because of an aspergilloma. Patients presenting with recurrent hemoptysis who have failed BAE should be assessed for surgical intervention.*
4. *The 5-year mortality rate for CCPA varies widely, ranging from 38% to 80%, with higher mortality observed in immunocompetent patients who also have an aspergilloma.*
5. *Aspergilloma and CCPA lie along the spectrum of chronic pulmonary aspergillosis, differentiated by the extent of structural lung destruction and clinical symptoms.*

## Financial/Nonfinancial Disclosures

None declared.
